# Dominant-Negative Effect of a Missense Variant in the TASK-2 (*KCNK5*) K^+^ Channel Associated with Balkan Endemic Nephropathy

**DOI:** 10.1371/journal.pone.0156456

**Published:** 2016-05-26

**Authors:** Alan P. Reed, Giovanna Bucci, Firdaus Abd-Wahab, Stephen J. Tucker

**Affiliations:** 1 Clarendon Laboratory, Department of Physics, University of Oxford, Oxford, United Kingdom; 2 OXION Initiative in Ion Channels and Disease, University of Oxford, Oxford, United Kingdom; Vanderbilt University Medical Center, UNITED STATES

## Abstract

TASK-2, a member of the Two-Pore Domain (K2P) subfamily of K^+^ channels, is encoded by the *KCNK5* gene. The channel is expressed primarily in renal epithelial tissues and a potentially deleterious missense variant in *KCNK5* has recently been shown to be prevalent amongst patients predisposed to the development of Balkan Endemic Nephropathy (BEN), a chronic tubulointerstitial renal disease of unknown etiology. In this study we show that this variant (T108P) results in a complete loss of channel function and is associated with a major reduction in TASK-2 channel subunits at the cell surface. Furthermore, these mutant subunits have a suppressive or ‘dominant-negative’ effect on channel function when coexpressed with wild-type subunits. This missense variant is located at the extracellular surface of the M2 transmembrane helix and by using a combination of structural modelling and further functional analysis we also show that this highly-conserved threonine residue is critical for the correct function of other K2P channels. These results therefore provide further structural and functional insights into the possible pathophysiological effects of this missense variant in TASK-2.

## Introduction

Balkan Endemic Nephropathy (BEN) is a hereditary, chronic renal disease occurring in several countries of the Balkan Peninsula [1]. The pathology of BEN is generally characterized by a slowly progressive atrophy and sclerosis of kidney structures thereby leading to end-stage renal failure, and consequently shares many similarities with several tubulointerstitial kidney diseases [2]. BEN is also frequently associated with upper urothelial cancer and this papillar carcinoma is often the most common cause of death in BEN patients [3]. Although many possible causes of the disease have been reported, including genetic predisposition, familial deficiency of enzymatic activity, genetic polymorphisms, and chromosomal aberrations [2, 4], the mechanisms leading to the development of this disease are still unknown [5].

Recently, a missense variant in the *KCNK5* gene that encodes the TASK-2 K2P K^+^ channel has been reported to exist in higher frequency amongst certain patients predisposed to BEN [6]. Potassium channels play a fundamentally important role in a renal function; in particular, they generate and maintain the resting membrane potential, regulate the contractility of renal vascular cells in the glomerulus, maintain potassium homeostasis and also facilitate both sodium reabsorption and many sodium-coupled transport processes [7]. In the human kidney, the TASK-2 channel has been shown to be highly expressed in the nephron [8], especially in tubular epithelia where the highly alkaline pH levels may play an important role in their regulation. More specifically, TASK-2 is thought to function as a molecular switch that modulates the epithelial K^+^ conductance relative to the rate of HCO_3_^−^ absorption [9,10]. Further evidence for the role that the TASK-2 channel plays in renal function comes from studies of a *KCNK5* null mouse model which exhibits significant renal dysfunction similar to that observed in human proximal renal tubular acidosis [11].

The variant in the *KCNK5* gene recently reported in BEN patients was one of three variants in different genes that were identified by exome sequencing of affected individuals [6]. This *KCNK5* variant (c.1397A>C) is predicted to change a threonine residue in the second transmembrane helix (M2) with a proline (T108P). The introduction of proline residues into α-helical transmembrane segments is well known to cause structural changes, and movement of the TM-helices is also known to play an important role in K^+^ channel function. Thus, given the reported role of TASK-2 channels in renal function, it is reasonable to assume that altered function of this gene might predispose certain patients towards this disease, and it has been reported that expression of this mutant in mammalian tsA201 cells produces a loss of function consistent with its proposed pathophysiological role [12]. In this study we have further characterized the structural and functional effects of this variant, and demonstrate that this mutation also exhibits a dominant-negative effect that will likely exacerbate the severely deleterious effect of this variant on normal TASK-2 channel function.

## Materials and Methods

### Molecular Biology

The wild-type human TASK-2 gene (*KCNK5*, Accession number: AF084830) was obtained from Source Bioscience and subcloned into a plasmid vector (pBF) suitable for *in vitro* transcription and expression in *Xenopus* oocytes. The T108P mutation was introduced by site-directed mutagenesis and mRNA was transcribed after vector linearization using SP6 RNA polymerase and the AmpliCap SP6 High Yield Message Maker kit (CellScript). The wild-type human TASK-2 gene and its variant T108P were also subcloned into pFAW-Ac-GFP vector, which adds GFP protein to the C-terminus of the inserted genes. For this vector the AmpliCap T7 High Yield Message Maker kit (CellScript) was used for transcription. mRNA was quantified using a Nanodrop spectrophotometer and mRNA integrity was assessed by agarose gel electrophoresis. Unless otherwise stated, a volume of 50 nl of mRNA was injected into Stage V and Stage VI *Xenopus* oocytes at a concentration of 50 ng μl^-1^ for either wild-type or mutant subunits (i.e. 2.5 ng per oocyte). For coinjection of WT and mutant mRNA at a 1:1 ratio, 2.5 ng of WT mRNA and 2.5 ng of mutant mRNA were injected per oocyte (or 2.5 ng of WT and 12.5 ng of mutant mRNA for the 1:5 ratio). Identical ratios/quantities mRNA for a non-functional channel subunit which does not coassemble with K2P channels (Kir2.1 G168X) [13] were also used as a control without effect. Oocytes were incubated for 18–24 h at 17°C in ND96 buffer at pH 7.4 (96 mM NaCl, 2 mM KCl, 2 mM MgCl2, 1.8 mM CaCl2, 5 mM HEPES). 2.5 mM sodium pyruvate, 0.05 mg/ml gentamicin and tetracycline, 0.1 mg/ml amikacin and ciprofloxacin were also added to the ND96 solution for oocyte culture.

### Electrophysiology

Two-electrode voltage clamp was used to record whole-cell currents using a GeneClamp 500 amplifier (Axon Instruments), Digidata 1322A (Axon Instruments) interface and pClamp 9.2 (Axon Instruments). All cells were tested at room temperature while being continuously perfused with ND96 bath solution (containing 96 mM NaCl, 2 mM KCl, 1.8 mM CaCl2, 2 mM MgCl2 and 10 mM HEPES) *via* a peristaltic pump perfusion system. Unless otherwise stated, currents were recorded using 300 ms voltage steps from a holding potential of -80 mV delivered in 20 mV increments between -140 mV and +100 mV. All recorded traces were analyzed using Clampfit (Axon Instruments), and graphs were plotted using Origin (OriginLab Corporation).

### Microscopy

Confocal fluorescence microscopy was used to determine the localization of either hTASK-2-GFP WT or hTASK-2-GFP T108P constructs expressed in *Xenopus* oocytes. All imaging was performed at room temperature using a PlanApochromat 63x /1.4 oil DIC objective. Cells were mounted on a chamber slide and viewed with a Zeiss LSM 510 META laser scanning system. The oocytes selected for confocal imaging had uniform animal and vegetal poles. Staining was performed by incubating the cells for 5 min in PBS containing 5ug/ml of CF 633-labeled wheat germ agglutinin (WGA-CF 633; Biotium), which stained the cells membrane from the extracellular side. The fluorescence signal originating from GFP-tagged proteins was identified using excitation with the 488 nm line of an argon laser. WGA-CF was excited by the 633 nm He-Ne laser. Emissions were collected at 530 nm and 660 nm.

## Results

### T108P variant results in a loss of TASK-2 channel function

**Fig 1:**
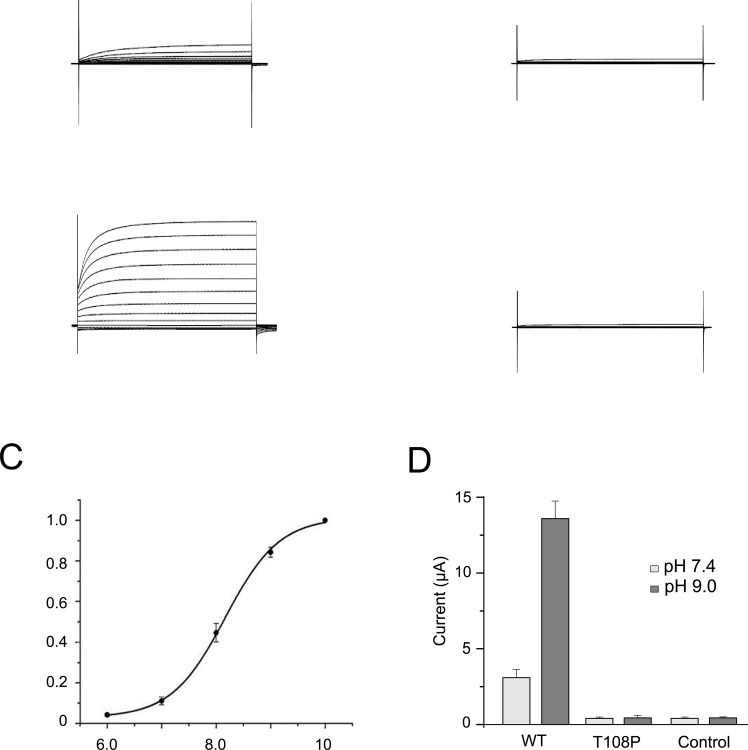
T108P variant in TASK-2 causes a loss of function. (A) Representative whole-cell current traces at physiological extracellular pH 7.4 recorded from oocytes injected with equivalent amounts of mRNA for either WT TASK-2 or the T108P variant. Currents were recorded using 300 ms voltage steps from a holding potential of -80 mV delivered in 20 mV increments between -140 mV and +100 mV. (B) Similar currents recorded after extracellular. (C) Activation of WT TASK-2 currents at alkaline pH. Results shown as means ± s.e.m. (D) Averaged whole-cell currents from uninjected control oocytes and cells expressing either WT or T108P TASK-2 channels at the indicated external pH values (WT *vs* T108P, *P<*0.01 at pH 7.4 and pH 9, one-way ANOVA, *post-hoc* Tukey HSD test; n = 9 for all conditions).

To understand the possible pathophysiological effect of this mutation we first measured the functional properties of the wild-type (WT) human TASK-2 channel heterologously expressed in *Xenopus* oocytes. Whole-cell currents were recorded by two-electrode voltage clamp from cells injected with WT TASK-2 mRNA (50 ng μl^−1^). These currents displayed an outwardly rectifying behavior in physiological solutions at pH 7.4 (3.53 ± 0.91 μA, at +100 mV, n = 9), similar to those previously reported for this channel [8]. However, by marked contrast, oocytes injected with similar amounts of mRNA for T108P mutant channel failed to produce any detectable K^+^ conductance; the observed currents (0.61 ± 0.11 μA, at +100 mV, n = 8) (Fig 1A), were indistinguishable from the background currents recorded from uninjected oocytes (0.93 ± 0.11 μA, at 100 mV, n = 9). We also found that injection of much higher concentrations of mRNA for this mutant (up to 600 ng μl^−1^) repeatedly failed to produce any detectable K^+^ currents (not shown). This result is consistent with a previous report of a loss of function [12] and confirms that this variant produces a severe loss of TASK-2 channel function in more than one expression system.

### Effects on activation by external pH

Anomalous titration of an arginine residue (R224) has been shown to be important for the increase in TASK-2 channel activity seen at alkaline pH. This residue is thought to modify the structural stability of the selectivity filter gating mechanism [14] and is in a similar position that of the T108P mutation, i.e. it is located close to the extracellular surface of one of the pore-lining helices (M4). We therefore examined whether the loss of function observed for the T108P mutation might be due to an ability of the channel to be activated by extracellular acidification.

To address this, we first examined the activation of wild-type TASK-2 channels by external alkalinization (Fig 1B and 1C). In agreement with previous reports, the activity of these WT channels (recorded at +100 mV) increased markedly at more alkaline external pH (pH_o_). (Fig 1B). At pH_o_ 9.0, WT TASK-2 currents increased >3-fold (Fig 1C and 1D). However, no increase in basal currents was observed for T108P mutant channels at pH_o_ 9.0 (Fig 1B and 1D) suggesting that, under these conditions, this mutation produces completely non-functional TASK-2 channels.

### Dominant-negative effect of the T108P mutation

**Fig 2:**
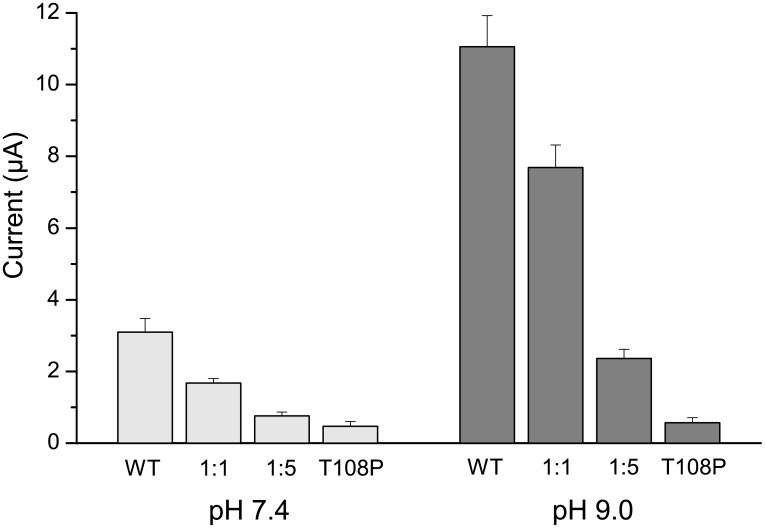
Dominant-negative effect of the T108P variant. Oocytes were coinjected with both WT TASK-2 and T108P mutant mRNA at different ratios and currents recorded at the indicated extracellular pH. At a 1:1 (WT:T108P) ratio the currents were markedly reduced. The effect is dose-dependent with further reductions at a ratio of 1:5 (WT:T108P). Results shown are means ± s.e.m. (WT *vs* 1:1, *P*<0.01 at pH 7.4 and pH 9; 1:1 vs 1:5, *P*<0.05 at pH 7.4 and pH9; 1:5 vs T108P, not significant at pH 7.4 or pH9, one-way ANOVA, *post-hoc* Tukey HSD test; n = 9 for all conditions).

K2P channel subunits contain two pore-forming units and assemble as a dimer to create a pseudo-tetrameric pore. Like classical tetrameric K^+^ channels they are therefore susceptible to the ‘dominant-negative effect’ that occurs in the heterozygous state where both wild-type and mutant subunits are coexpressed, and where coassembly of mutant subunits into the pore affects the functional properties of the channel [15, 16]. Most patients identified with this mutation are heterozygous [6] and so we therefore assessed the ability of this variant to influence WT TASK-2 channel function by coinjecting mRNA for WT and mutant subunits. At physiological pH_o_ 7.4, co-expression of WT and T108P mutant subunits in a 1:1 ratio caused a reduction of WT current amplitude by >40% (Fig 2). This effect was dose-dependent and injection of a 1:5 ratio (WT:T108P) reduced currents to near background levels. Dose-dependent dominant-negative effects were also observed at pH_o_ 9.0 (Fig 2). As a control, and similar to that shown before [15,16], no reduction in current was observed if coinjected with identical quantities of an unrelated mRNA (not shown). Overall, these results demonstrate that T108P subunits are able to coassemble with WT TASK-2 subunits to down-regulate channel activity in the heterozygous state.

### Structural modelling of the T108P mutation

**Fig 3:**
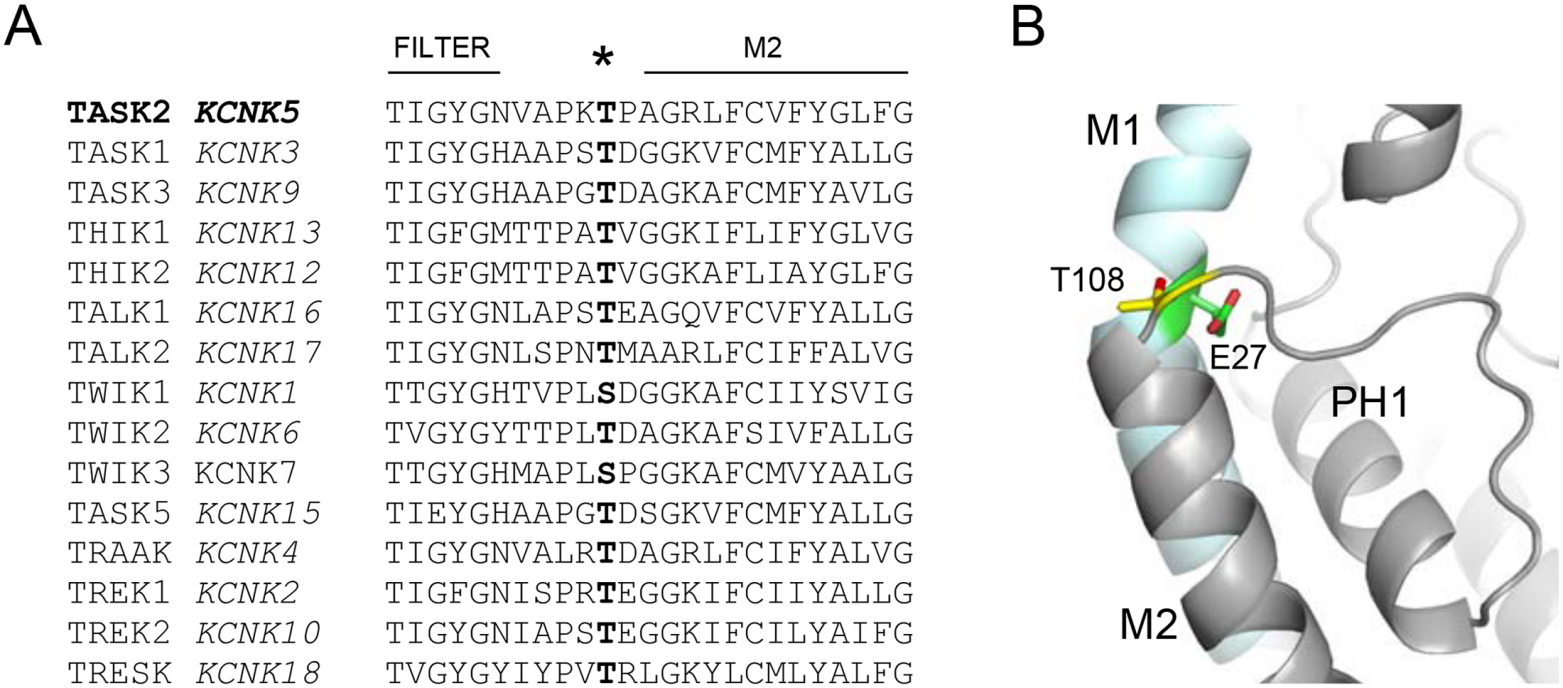
Structural modelling of the T108P variant. (A) Amino acid sequence alignment of the selectivity filter to M2 region containing the T108P variant for all 15 members of the human K2P family of K^+^ channels showing the highly-conserved nature of the mutated residue. T108 is indicated by an asterisk. (B) Homology model of TASK-2 created using the crystal structure of TREK-2. This reveals that T108 is located at the top of the M2 helix in the loop that connects M2 to the selectivity filter. T108 (yellow) on M2 hydrogen bonds with the backbone and side chain of E27 (green) located on the adjacent M1 helix (cyan).

Comparison of amino acid sequences for the 15 known human K2P channels reveals that this threonine residue is TASK-2 is almost completely conserved across this family except two members of the TWIK subfamily (*KCNK1* and *KCNK7*) that have a serine at this position (Fig 3A). This high degree of sequence conservation indicates that this residue may be important for K2P channel function. We therefore created a homology model of TASK-2 based upon the available crystal structure of the related human TREK-2 channel (PDB: 4XDJ) [17].

This homology model predicts that this mutated residue is located at the extracellular face of the channel, just before the M2 α-helix begins (Fig 3B). The model also predicts that the threonine side-chain forms a hydrogen bond to the backbone of another highly conserved glutamate residue (E27) on the adjacent M1 helix. Intriguingly, mutation of this glutamate has been shown to disrupt the pH-sensing mechanism in other K2P channels [18]. Furthermore, the high degree of sequence conservation at this site suggests that H-bonding between M2 and M1 at this position may be feature in all K2P channels. However, as observed with this particular missense variant, mutation of this residue to a proline is likely to have a major impact on the structural flexibility of this loop and will not just affect H-bonding at this site.

### Effect of an equivalent mutation in TREK-1

To further probe the importance of this highly-conserved residue in K2P channel function we mutated the equivalent residue (T167P) in the well-characterized human TREK-1 channel [10]. At physiological pH_o_, oocytes expressing this mutant produced background level currents which did not appear different from uninjected oocytes (TREK-1 T167P: 0.72 ± 0.04 μA, n = 8; uninjected oocytes, 0.78 ± 0.12 μA, n = 7). Injection of 5-fold higher concentration of mRNA (100 ng μl^−1^) also failed to produce functional channels (not shown).

We also attempted activation of these background currents with BL-1249, a compound known to dramatically enhance WT TREK-1 currents [19]. Interestingly, although BL-1249 (30 μM) activated WT TREK-1 currents by >5 fold, it had no effect on the TREK-1 T167P mutant. TREK-1 is also activated by external alkalinization [10], but we found that pH 9.0 failed to activate the TREK-1 T167P mutant (not shown). Overall, these results support our observation that mutation of this highly conserved residue dramatically impacts K2P channel function.

### Systematic mutagenesis of Threonine 108

**Fig 4:**
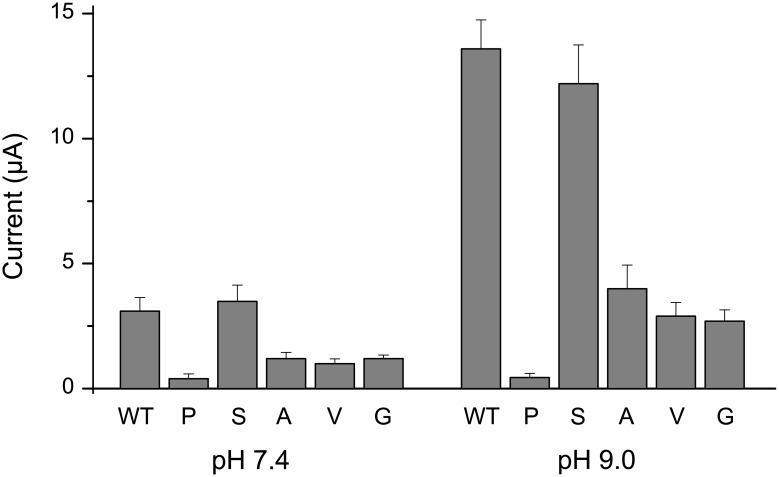
Effect of different amino-acid mutations at T108. Summary of mean currents recorded at different external pH values for oocytes injected with either WT TASK-2 or the indicated amino-acid mutations at T108. In comparison to WT, current amplitudes were markedly reduced for all mutants except T108S suggesting that H-bonding at this position is required for correct channel function. Results are shown as means ± s.e.m (WT *vs* T108P/A/V/G, *P*<0.01 at pH 7.4 and pH 9; T108P *vs* T108V, *P*<0.05 at pH 7.5 and pH 9; T108P *vs* T108G and T108A, *P*<0.01 at pH 7.5, *P*<0.05 at pH 9, one-way ANOVA, *post-hoc* Tukey HSD test; n = 9 for all conditions).

The principal gating mechanism in K2P channels is thought to located within the selectivity filter [20–23] and the flexibility of the external loops that connect to this region are thought to play a critical role in the regulation of channel gating [10]. The predicted H-bonding between T108 at the top of M2 and the highly conserved glutamate (E27) at the top of M1 may be important for correct channel function and it has also been shown that mutation of this glutamate produces non-functional channels [24]. Therefore, to investigate the possible structural reasons underlying the loss of function by the T108P mutation we engineered TASK-2 variants with different amino acid substitutions at this position (Fig 4).

Interestingly, we found that the T108S mutant exhibited properties almost identical to WT TASK-2, whereas the T108A, T108V and T108G mutations all resulted in markedly reduced currents at physiological pH. However, unlike the T108P mutant, these currents all increased upon extracellular alkalinization, although to a lesser extent than WT TASK-2 channel (Fig 4). These results suggest that the presence of a hydroxyl group in the threonine and serine side chains is essential for correct channel function, and that the proline mutation may have additional effects on channel function unrelated to the loss of H-bonding at this position.

### The T108P mutation reduces cell surface expression

Mutation of highly conserved residues can have effects on protein structure and folding, as well as protein function. In the case of membrane proteins such misfolding can also result in their retention in the endoplasmic reticulum (ER) and/or subsequent degradation [25, 26]. We therefore investigated whether the apparent major loss of function observed for the T108P mutation was due to a reduced number of mutant subunits in the cell membrane.

**Fig 5:**
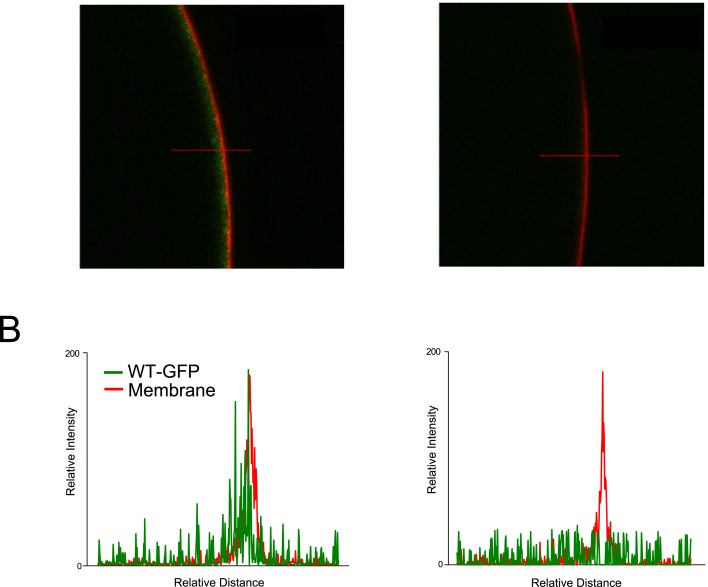
T108P reduces trafficking to the cell membrane. Confocal microscopy of GFP-tagged WT and mutant TASK-2 channels. (A) WT TASK-2 and T108P tagged with GFP at the C-termini expressed in oocytes. The red fluorescent signal (Wheat Germ Agglutinin CF633) indicates the location of the cell membrane. WT channels tagged with GFP (green) exhibit a clear membrane-associated fluorescence, whereas the mutant T108P channels showed no membrane localization, and no GFP fluorescence in any other part of the oocyte. (B) Representative relative signal-intensity profiles for oocytes expressing WT or T108P mutant channels. Intensities were determined along the cross-sections indicated by the red lines in panel A.

To monitor this we fused green fluorescent protein (GFP) to the C-terminus of both WT and T108P mutant TASK-2 channels, expressed them in oocytes and measured their relative surface expression using confocal fluorescence microscopy. Importantly, fusion of GFP to the C-terminus did not appear to affect the functional properties of TASK-2; these tagged subunits produced whole cell currents similar in size to that of WT TASK-2 that could be activated at alkaline pH. Consistent with their functional expression, a strong membrane-localized fluorescence could also be observed. This was in marked contrast to the T108P-GFP mutant channel where no localization near the cell perimeter could be observed (Fig 5). The levels of fluorescence observed for the mutant channel were similar to those measured for uninjected oocytes under the same conditions (not shown). Overall, these results strongly suggest that the T108P mutation also disturbs the correct assembly/processing/trafficking of these mutant subunits so that fewer channels appear at the cell surface.

## Discussion

In this study we have shown that the *KCNK5* missense variant (T108P) associated with Balkan Endemic Nephropathy results in a complete loss of function in homomeric TASK-2 channels when expressed in *Xenopus* oocytes, and that there is impaired trafficking and/or processing of these mutant subunits to the cell surface. Furthermore, we show that these mutant subunits also have a major suppressive or ‘dominant-negative’ effect on channel function when coexpressed with wild-type subunits. Structural modelling and further functional analysis also reveals that H-bonding between this highly conserved threonine residue on M2 and E27 on the M1 helix appears to be critical for correct K2P channel function. These results therefore provide further structural and functional insight into the possible pathophysiological effects of this missense variant in the TASK-2 K2P channel.

Increasing knowledge of the TASK/TALK subfamily of K2P channels reveals that they play an important role in the regulation of both renal and respiratory physiology [27, 28]. This is perhaps not surprising given the interplay between both systems in acid-base balance. Such homeostasis is achieved primarily by regulating bicarbonate exchange (mainly in the proximal tubule) and the secretion of buffered protons (in the distal tubule) where members of the TASK subfamily, in particular TASK-2, play an important role in this process [28]. Due to their extracellular pH-sensitivity, TASK-2 channels are also thought to play a critical role in the regulation of CO_2_/H^+^ levels in the CNS and to tune respiratory activity. Therefore the idea that a missense variant in the *KCNK5* gene encoding the TASK-2 channel may predispose certain patients towards BEN is clearly strengthened by the important role that this channel plays in renal physiology [6].

The complete loss of function we observe for this variant may arise from modified gating properties of the channel, reduced numbers at the cell surface or more likely, a combination of both factors. Our results suggest that only side chains capable of H-bonding at this position (i.e. serine and threonine) lead to correct channel function and therefore mutation to a proline residue will prevent this interaction. However, the results also suggest that the complete loss of function seen with the T108P mutation may result from reduced numbers of TASK-2 channels in the cell membrane. Compared to the other mutations we tested, the introduction of a proline at this position is likely to have a greater effect on the structure of the channel at a position where it begins to form the M2 α-helix. This would therefore not only reduce H-bonding at this position, but might also affect protein folding, insertion into the membrane and trafficking to the cell membrane. Such mutant proteins are therefore unlikely to escape the general quality control mechanisms in the ER leading to their retention and/or degradation [25, 26].

Non-functional ion channel subunits are also known to produce a ‘dominant-negative’ effect in the heterozygous state by coassembly with WT subunits, although due to their dimeric assembly, the extent of the dominant-negative effect in K2P channels can sometimes be less pronounced than for classical tetrameric channels [15, 16, 26]. Nevertheless, this down regulation of channel activity will either result from incorrect assembly and degradation of heteromeric channels, or their dysfunction [15, 16, 26]. In the case of the T108P variant, the fact that the homomeric channels do not reach the membrane may be one of the main reasons underlying this dominant-negative effect. However, it remains unclear whether the suppressive effect of this mutation is primarily due to reduced heteromeric channel function, or a failure to reach the cell surface, or perhaps reasons. Further experiments would be required to establish the importance of these different mechanisms and expression in *Xenopus* oocytes may not be the best system to address such detailed questions about changes in intracellular trafficking and cell biology. Nevertheless, this loss of function phenotype for the T108P mutation is unlikely to be an artefact of the oocyte expression system because a similar loss of function has also been observed when this variant is expressed as a homomeric channel in mammalian tsA201 cells [12].

Loss-of-function mutations in potassium channels have been linked to many human channelopathies and although mutations in K2P channels appear to be observed less frequently than those in e.g. voltage-gated or inward rectifier K^+^ channels, a number of associated channelopathies are beginning to emerge. For example, loss of function mutations in TASK-3 are associated with Birk-Barel Syndrome [29], mutations in TWIK-2 and TASK-1 with pulmonary hypertension [30, 31] and mutations in the TRESK channel with certain forms of migraine [15, 16]. In the latter case, loss of function variants in TRESK were also found to operate in a similar dominant-negative fashion, although the precise nature of the heterologous expression system appears to influence the severity of this effect [15, 32]. Likewise, in the case of Birk-Barel syndrome, incomplete penetrance of the allele and other epigenetic factors are also thought to influence the impact of the mutation [29].

The etiology of BEN is complex and any genetic predisposition is also likely to be complex. Nevertheless, our results suggest that any patient who possesses this missense variant is likely to have severely impaired TASK-2 channel function whether they are homo- or heterozygous for this mutation. The ‘loss of function’ associated with this variant is severe and therefore clearly has the potential to impact renal function. It seems remarkable that patients with such a severe functional mutation do not exhibit a much clearer phenotype, but several studies have now demonstrated that apparently ‘healthy’ individuals within the population can harbor a number of complete loss of function variants in supposedly ‘essential’ genes without adverse effects [33, 34].

In summary, this study demonstrates that the T108P mutation in TASK-2 results in a severe loss of channel function and examines the structural and functional basis for this effect. This therefore highlights the potential pathophysiological role that this variant may play in the declining renal function of patients with BEN and may help with better diagnosis of this complex disorder.

## References

[1] Pavlovic NM (2013) Balkan endemic nephropathy-current status and future perspectives. Clin Kidney J 6: 257–265. 10.1093/ckj/sft049 26064484 PMC4400492

[2] Toncheva D, Dimitrov T, Stojanova S (1998) Etiology of Balkan endemic nephropathy: a multifactorial disease? Eur J Epidemiol 14: 389–394. 9690758 10.1023/a:1007445120729

[3] Stefanovic V, Polenakovic M, Toncheva D (2011) Urothelial carcinoma associated with Balkan endemic nephropathy; a worldwide disease. Pathol Biol (Paris) 59: 286–291. 19896305 10.1016/j.patbio.2009.05.002

[4] Toncheva D, Dimitrov T (1996) Genetic predisposition to Balkan endemic nephropathy. Nephron 72: 564–569. 8730422 10.1159/000188940

[5] Stefanovic V, Toncheva D, Polenakovic M (2015) Balkan nephropathy. Clin Nephrol 83: 64–69. 25725245 10.5414/cnp83s064

[6] Toncheva D, Mihailova-Hristova M, Vazharova R, Staneva R, Karachanak S, Dimitrov P, et al (2014) NGS nominated *CELA1*, *HSPG2*, and *KCNK5* as candidate genes for predisposition to Balkan endemic nephropathy. Biomed Res Int 2014: 920723 10.1155/2014/920723 24949484 PMC4052113

[7] Wang W, Hebert SC, Giebisch G (1997) Renal K^+^ channels: structure and function. Annu Rev Physiol 59: 413–436. 9074771 10.1146/annurev.physiol.59.1.413

[8] Reyes R, Duprat F, Lesage F, Fink M, Salinas M, Farman N, et al (1998) Cloning and expression of a novel pH-sensitive two pore domain K^+^ channel from human kidney. J Biol Chem 273: 30863–30869. 9812978 10.1074/jbc.273.47.30863

[9] Lesage F, Barhanin J (2011) Molecular physiology of pH-sensitive background K2P channels. Physiology (Bethesda) 26: 424–437. 22170960 10.1152/physiol.00029.2011

[10] Sepulveda FV, Pablo Cid L, Teulon J, Niemeyer MI (2015) Molecular aspects of structure, gating, and physiology of pH-sensitive background K2P and Kir K^+^-transport channels. Physiol Rev 95: 179–217. 10.1152/physrev.00016.2014 25540142 PMC4281587

[11] Warth R, Barriere H, Meneton P, Bloch M, Thomas J, Tauc M, et al (2004) Proximal renal tubular acidosis in TASK2 K^+^ channel-deficient mice reveals a mechanism for stabilizing bicarbonate transport. Proc Natl Acad Sci U S A 101: 8215–8220. 15141089 10.1073/pnas.0400081101 PMC419583

[12] Veale EL, Mathie A (2016) Aristolochic acid, a plant extract used in the treatment of pain and linked to Balkan Endemic Nephropathy, is a regulator of K2P channels. Br J Pharmacol 173:1639–52 10.1111/bph.13465 26914156 PMC4842925

[13] Nagaoka Y, Shang L, Banerjee A, Bayley H, Tucker SJ (2008) Peptide backbone mutagenesis of putative gating hinges in a potassium ion channel. ChemBiochem 9: 1725–1728. 10.1002/cbic.200800133 18543260

[14] Lopez-Cayuqueo KI, Pena-Munzenmayer G, Niemeyer MI, Sepulveda FV, Cid LP (2015) TASK-2 K2P K^+^ channel: thoughts about gating and its fitness to physiological function. Pflugers Arch 467: 1043–1053. 10.1007/s00424-014-1627-7 25315981

[15] Andres-Enguix I, Shang L, Stansfeld PJ, Morahan JM, Sansom MS, et al (2012) Functional analysis of missense variants in the TRESK (KCNK18) K channel. Sci Rep 2: 237 10.1038/srep00237 22355750 PMC3266952

[16] Lafreniere RG, Cader MZ, Poulin JF, Andres-Enguix I, Simoneau M, Lafrenière RG, et al (2010) A dominant-negative mutation in the TRESK potassium channel is linked to familial migraine with aura. Nat Med 16: 1157–1160. 10.1038/nm.2216 20871611

[17] Dong YY, Pike AC, Mackenzie A, McClenaghan C, Aryal P, Dong L, et al (2015) K2P channel gating mechanisms revealed by structures of TREK-2 and a complex with Prozac. Science 347: 1256–1259. 10.1126/science.1261512 25766236 PMC6034649

[18] Cohen A, Ben-Abu Y, Hen S, Zilberberg N (2008) A novel mechanism for human K2P2.1 channel gating. Facilitation of C-type gating by protonation of extracellular histidine residues. J Biol Chem 283: 19448–19455. 10.1074/jbc.M801273200 18474599

[19] Veale EL, Al-Moubarak E, Bajaria N, Omoto K, Cao L, Tucker SJ, et al (2014) Influence of the N terminus on the biophysical properties and pharmacology of TREK1 potassium channels. Mol Pharmacol 85: 671–681. 10.1124/mol.113.091199 24509840

[20] Piechotta PL, Rapedius M, Stansfeld PJ, Bollepalli MK, Ehrlich G, Andres-Enguix I, et al (2011) The pore structure and gating mechanism of K2P channels. EMBO J 30: 3607–3619. 10.1038/emboj.2011.268 21822218 PMC3181484

[21] Schewe M, Nematian-Ardestani E, Sun H, Musinszki M, Cordeiro S, Bucci G, et al (2016) A Non-canonical Voltage-Sensing Mechanism Controls Gating in K2P K^+^ Channels. Cell 164: 937–949. 10.1016/j.cell.2016.02.002 26919430 PMC4771873

[22] Zilberberg N, Ilan N, Goldstein SA (2001) KCNKO: opening and closing the 2-P-domain potassium leak channel entails "C-type" gating of the outer pore. Neuron 32: 635–648. 11719204 10.1016/s0896-6273(01)00503-7

[23] Rapedius M, Schmidt MR, Sharma C, Stansfeld PJ, Sansom MS, Baukrowitz T, et al (2012) State-independent intracellular access of quaternary ammonium blockers to the pore of TREK-1. Channels (Austin) 6: 473–478. 22991046 10.4161/chan.22153 PMC3536734

[24] Morton MJ, Abohamed A, Sivaprasadarao A, Hunter M (2005) pH sensing in the two-pore domain K^+^ channel, TASK2. Proc Natl Acad Sci U S A 102: 16102–16106. 16239344 10.1073/pnas.0506870102 PMC1276079

[25] Ma D, Jan LY (2002) ER transport signals and trafficking of potassium channels and receptors. Curr Opin Neurobiol 12: 287–292. 12049935 10.1016/s0959-4388(02)00319-7

[26] Tucker SJ, Bond CT, Herson P, Pessia M, Adelman JP (1996) Inhibitory interactions between two inward rectifier K^+^ channel subunits mediated by the transmembrane domains. J Biol Chem 271: 5866–5870. 8621458 10.1074/jbc.271.10.5866

[27] Bayliss DA, Barhanin J, Gestreau C, Guyenet PG (2015) The role of pH-sensitive TASK channels in central respiratory chemoreception. Pflugers Arch 467: 917–929. 10.1007/s00424-014-1633-9 25346157 PMC4400208

[28] Gestreau C, Heitzmann D, Thomas J, Dubreuil V, Bandulik S, Reichold M, et al (2010) Task2 potassium channels set central respiratory CO_2_ and O_2_ sensitivity. Proc Natl Acad Sci U S A 107: 2325–2330. 10.1073/pnas.0910059107 20133877 PMC2836670

[29] Barel O, Shalev SA, Ofir R, Cohen A, Zlotogora J, Shorer Z, et al (2008) Maternally inherited Birk Barel mental retardation dysmorphism syndrome caused by a mutation in the genomically imprinted potassium channel *KCNK9*. Am J Hum Genet 83: 193–199. 10.1016/j.ajhg.2008.07.010 18678320 PMC2495061

[30] Pathan AR, Rusch NJ (2011) Two-pore domain K^+^ channels: evidence for TWIK-2 in blood pressure regulation. Hypertension 58: 539–541. 10.1161/HYPERTENSIONAHA.111.179390 21876073

[31] Antigny F, Hautefort A, Meloche J, Belacel-Ouari M, Manoury B, Rucker-Martin C, et al (2016) Potassium Channel Subfamily K Member 3 (*KCNK3*) Contributes to the Development of Pulmonary Arterial Hypertension. Circulation 133: 1371–1385. 10.1161/CIRCULATIONAHA.115.020951 26912814

[32] Guo Z, Liu P, Ren F, Cao YQ (2014) Nonmigraine-associated TRESK K^+^ channel variant C110R does not increase the excitability of trigeminal ganglion neurons. J Neurophysiol 112: 568–579. 10.1152/jn.00267.2014 24805079 PMC4122697

[33] MacArthur DG, Balasubramanian S, Frankish A, Huang N, Morris J, Walter K, et al (2012) A systematic survey of loss-of-function variants in human protein-coding genes. Science 335: 823–828. 10.1126/science.1215040 22344438 PMC3299548

[34] Narasimhan VM, Hunt KA, Mason D, Baker CL, Karczewski KJ, Barnes MR, et al (2016) Health and population effects of rare gene knockouts in adult humans with related parents. Science 352:474–7. 10.1126/science.aac8624 26940866 PMC4985238

