# Correction: Dominant-Negative Effect of a Missense Variant in the TASK-2 (*KCNK5*) K^+^ Channel Associated with Balkan Endemic Nephropathy

**DOI:** 10.1371/journal.pone.0160114

**Published:** 2016-07-21

**Authors:** Alan P. Reed, Giovanna Bucci, Firdaus Abd-Wahab, Stephen J. Tucker

Several labels are missing from Figs 1 and 5 images. Please see the corrected Figs 1 and 5 here.

**Fig 1:**
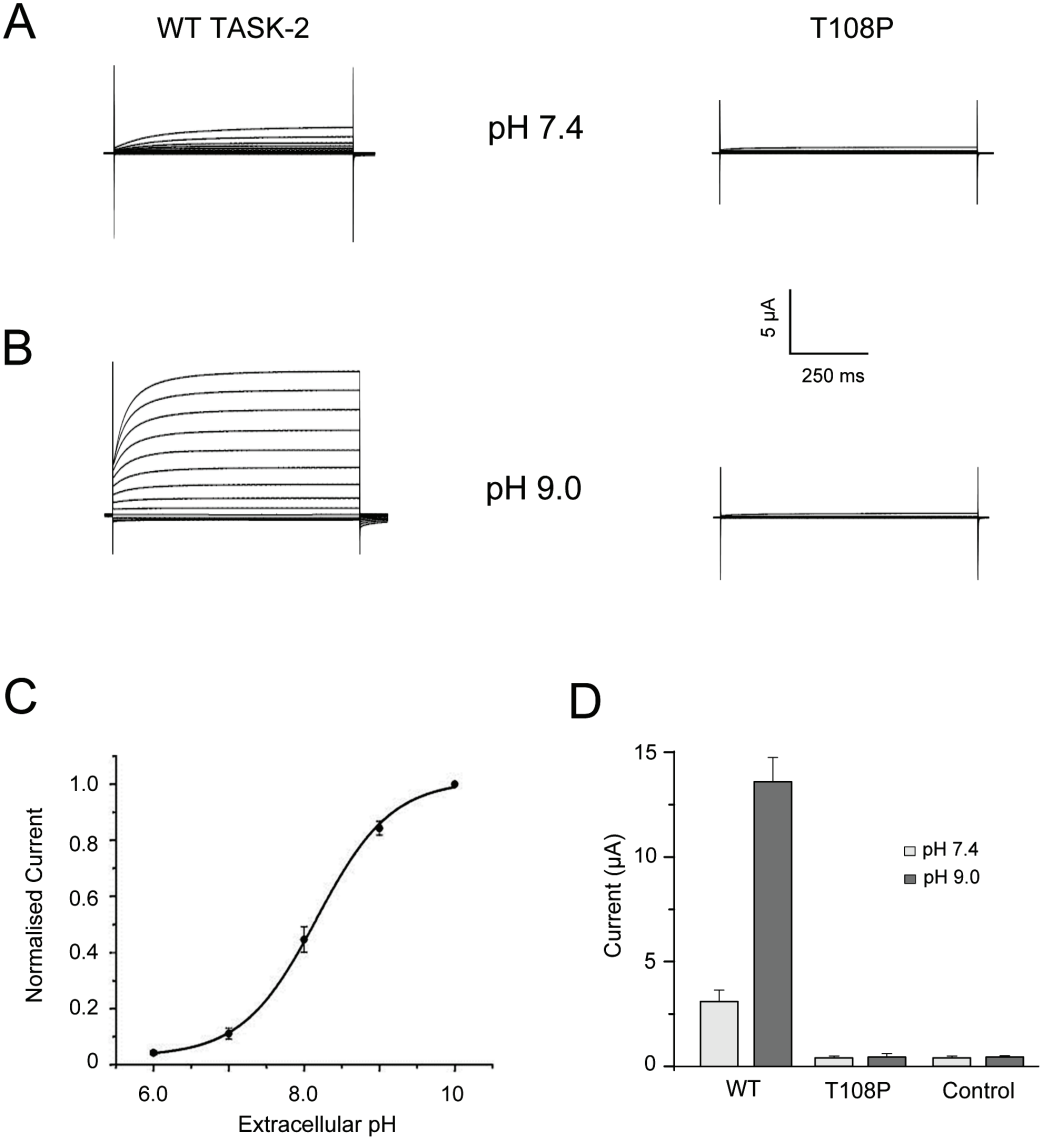
T108P variant in TASK-2 causes a loss of function. (A) Representative whole-cell current traces at physiological extracellular pH 7.4 recorded from oocytes injected with equivalent amounts of mRNA for either WT TASK-2 or the T108P variant. Currents were recorded using 300 ms voltage steps from a holding potential of -80 mV delivered in 20 mV increments between -140 mV and +100 mV. (B) Similar currents recorded after extracellular. (C) Activation of WT TASK-2 currents at alkaline pH. Results shown as means ± s.e.m. (D) Averaged whole-cell currents from uninjected control oocytes and cells expressing either WT or T108P TASK-2 channels at the indicated external pH values (WT *vs* T108P, *P<*0.01 at pH 7.4 and pH 9, one-way ANOVA, *post-hoc* Tukey HSD test; n = 9 for all conditions).

**Fig 5:**
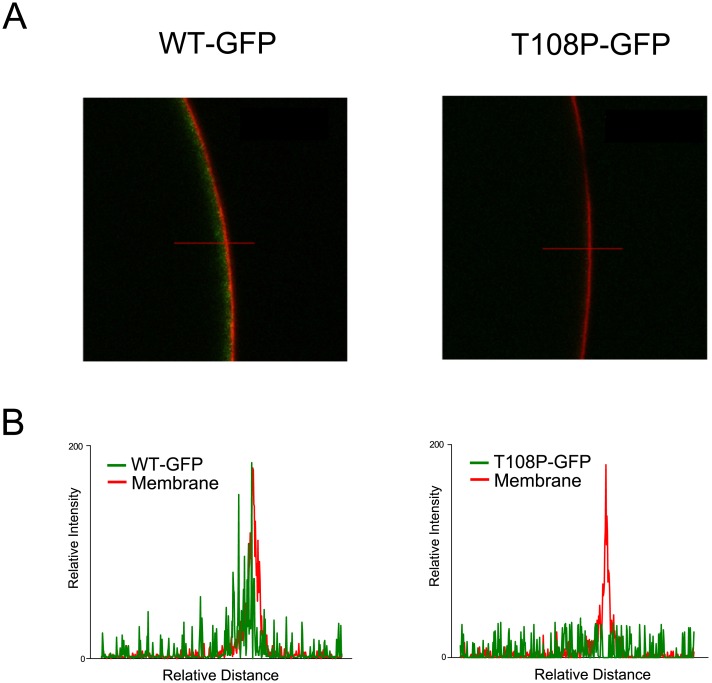
T108P reduces trafficking to the cell membrane. Confocal microscopy of GFP-tagged WT and mutant TASK-2 channels. (A) WT TASK-2 and T108P tagged with GFP at the C-termini expressed in oocytes. The red fluorescent signal (Wheat Germ Agglutinin CF633) indicates the location of the cell membrane. WT channels tagged with GFP (green) exhibit a clear membrane-associated fluorescence, whereas the mutant T108P channels showed no membrane localization, and no GFP fluorescence in any other part of the oocyte. (B) Representative relative signal-intensity profiles for oocytes expressing WT or T108P mutant channels. Intensities were determined along the cross-sections indicated by the red lines in panel A.
